# Group A streptococcal bacteremias in Southwest Finland 2007–2018: epidemiology and role of infectious diseases consultation in antibiotic treatment selection

**DOI:** 10.1007/s10096-020-03851-6

**Published:** 2020-02-25

**Authors:** Johanna Vilhonen, Jaana Vuopio, Tero Vahlberg, Kirsi Gröndahl-Yli-Hannuksela, Kaisu Rantakokko-Jalava, Jarmo Oksi

**Affiliations:** 1 Department of Infectious Diseases, Turku University Hospital; Doctoral Programme in Clinical Research (DPCR), University of Turku, Turku, Finland; 2 Institute of Biomedicine, University of Turku; Department of Clinical Microbiology, Turku University Hospital, Turku, Finland; 3 Department of Clinical Medicine, Biostatistics, University of Turku, Turku, Finland; 4 Institute of Biomedicine, University of Turku, Turku, Finland; 5 Department of Clinical Microbiology, Turku University Hospital, Turku, Finland; 6 Department of Infectious Diseases, Turku University Hospital, University of Turku, Turku, Finland

**Keywords:** Group A streptococcus, Epidemiology, Antibiotic treatment, Infectious diseases specialist consultation

## Abstract

The incidence of invasive group A streptococcal (GAS) infections has shown a fluctuating but increasing trend in Finland. The impact of infectious diseases specialist consultation (IDSC) on the antimicrobial therapy of GAS bacteremia has not been studied earlier. A retrospective study on adult GAS bacteremia in The Hospital District of Southwest Finland (HDSWF) was conducted from 2007 to 2018. Data on incidence of bacteremic GAS cases were gathered from the National Infectious Disease Register. Clinical data were obtained by reviewing the electronic patient records. The overall incidence of GAS bacteremia in HDSWF was 3.52/100,000, but year-to-year variation was observed with the highest incidence of 7.93/100,000 in 2018. A total of 212 adult GAS bacteremia cases were included. A record of IDSC was found (+) in 117 (55.2%) cases, not found (−) in 71 (33.5%) cases and data were not available in 24 (11.3%) cases. Among IDSC+ cases, 57.3% were on penicillin G treatment whereas in the group IDSC− only 22.5%, respectively (OR = 4.61, 95% CI 2.37–8.97; *p* < 0.001). The use of clindamycin as adjunctive antibiotic was more common among IDSC+ (54.7%) than IDSC− (21.7%) (OR = 4.51, 95% CI 2.29–8.87; *p* < 0.001). There was an increasing trend in incidence of GAS bacteremia during the study period. Narrow-spectrum beta-lactam antibiotics were chosen, and adjunctive clindamycin was more commonly used, if IDSC took place. This highlights the importance of availability of IDSC but calls for improved practice among infectious diseases specialists by avoiding combination therapy with clindamycin in non-severe invasive GAS infections.

## Introduction

*Streptococcus pyogenes* (group A streptococcus, GAS) is a well-recognized human pathogen that causes commonly non-invasive infections such as pharyngitis and non-necrotizing cellulitis but can also cause invasive infections (iGAS) such as bacteremia and severe iGAS infections, e.g., streptococcal toxic shock syndrome (STSS) and necrotizing fasciitis (NF), respectively. GAS infections are associated with remarkable morbidity and mortality worldwide with an estimated 500,000 deaths per year [1, 2].

The incidence of iGAS infections is known to fluctuate. In Finland, an increasing trend in the overall incidence has been observed since 1995 [3–5]. Concordant observations are reported also from other countries [6–8].

GAS isolates have remained universally susceptible to penicillin and penicillin-resistant strains have not been reported to date. However, Vannice et al. reported recently clinical GAS isolates with a *pbp2x* missense mutation and elevated minimum inhibitory concentrations for ampicillin, amoxicillin and cefotaxime [9]. Similarly, GAS strains with lowered susceptibility to beta-lactams have been reported in a large multicountry study, including Finland [10]. Beta-lactam antibiotics, and especially penicillin, are the basis of the antimicrobial therapy of GAS infections. Concurrent therapy with clindamycin is observed to reduce mortality in severe iGAS infections [11–13].

The Hospital District of Southwest Finland (HDSWF) has a total of over 470,000 residents and 5 hospitals (Turku University Hospital and regional hospitals in Salo, Loimaa, Uusikaupunki, and Turku). The aim of the study is to report incidence, *emm* type distribution, clinical pictures and outcome of GAS bacteremias in the HDSWF during 2007–2018. In addition, our purpose is to assess the effect of an infectious diseases specialist consultation (IDSC) on the antimicrobial therapy of GAS bacteremia during this study period.

## Materials and methods

### Data collection

Since 1995, invasive GAS infections (only isolations from blood and cerebrospinal fluid) have been notifiable in Finland according to the Communicable Diseases Act. The diagnosing laboratory reports GAS bacteremias and isolations from cerebrospinal fluid to the National Infectious Disease Register (NIDR) maintained by the Finnish institute of health and welfare (THL). The data of each iGAS case are recorded in the NIDR to the hospital district that the patient belongs to according to their place of domicile. Data on incidence of bacteremic GAS cases were gathered from NIDR [14].

The Department of Hospital Hygiene and Infection Control in the HDSWF maintains a database (SAI) to which all the microbial findings in clinical samples, e.g., bacteremias, are recorded with the national identity code of the patient and the date of specimen. The SAI database records all iGAS cases treated in HDSWF irrespective of patient’s place of domicile. All adult bacteremic GAS cases treated in the hospitals of HDSWF from Jan 2007 until Dec 2018 were retrospectively identified from SAI-register. The cases meeting the inclusion criteria were included in this study, and their electronic patient records were reviewed.

The electronic patient records were read by an infectious diseases specialist (JVi) and the data on patient demographics, underlying diseases, clinical course of bacteremia, infectious foci, antibiotic therapy, a record of IDSC and outcome were documented.

### Inclusion criteria

The inclusion criteria were at least one positive blood culture for GAS with concurrent clinical signs of infection, age of *≥* 18 years and at least one contact to any of the hospitals of the HDSWF during the GAS bacteremia episode.

### *emm* typing

THL has *emm*-typed invasive GAS strains since 2005, and data on *emm* types were acquired from NIDR. In altogether, 10 cases the data on *emm* types were not available from NIDR. For 8 cases of these 10, the isolate was obtained from the culture collection of Turku University Hospital and typed at the University of Turku. *Emm* typing was performed according to the guidelines of the Centers for Disease Control and Prevention (CDC) [15].

### Definitions

The underlying diseases were classified according to the Charlson Comorbidity Index (CCI). The CCI was further divided into four categories according to the Charlson’s original study, 0 score is 0, 1–2 scores is 1, 3–4 scores is 2 and ≥ 5 is 3 [16].

Healthcare-acquired GAS bacteremia was defined as (1) bacteremia with positive blood cultures for GAS gathered ≥ 48 h after hospital admission or (2) GAS bacteremia associated with a healthcare-related procedure (including labor and previous hospital discharge) during 30 days before the positive blood cultures for GAS or (3) GAS bacteremia associated with foreign body surgery during 90 days before the positive blood cultures for GAS.

The definition of STSS was according to the case definition 2010 of CDC and included identification of GAS from a normally sterile site, hypotension and at least two other organ failures [17].

The infectious diseases specialist consultation was defined as a written note of IDSC in the patient record done by the treating doctor or infectious disease specialist during 5 days after the positive blood cultures for GAS. No available data on consultation (NADC) was used for cases who died during the 48 h after the positive blood cultures for GAS or who were transferred to a health care unit outside of HDSWF before the answer of blood cultures were available.

The first-line antibiotics and adjunctive antibiotics were defined as antibiotics given to the patient at the time when the first positive GAS blood culture result was available until the fifth day after taking the blood cultures. Beta-lactam antibiotics and vancomycin were regarded as first-line antibiotics and other antibiotics, if used, adjunctive antibiotics.

### Statistical analyses

Statistical analyses were performed with the IBM SPSS Statistics for Windows version 25 (IBM Corp., Armonk, NY). A two-sided *p* < 0.05 was considered statistically significant. Categorical data were analyzed by chi-square test, and mean age between IDSC groups was tested by two-sample test. The differences in antibiotic treatment selections between groups were analyzed by binary logistic regression. Poisson regression analysis was used to examine the change in incidence of GAS bacteremia in HDSWF during the 4-year follow-up periods (2007–2010, 2011–2014, and 2015–2018).

## Results

### Epidemiology and clinical characteristics

**Fig. 1:**
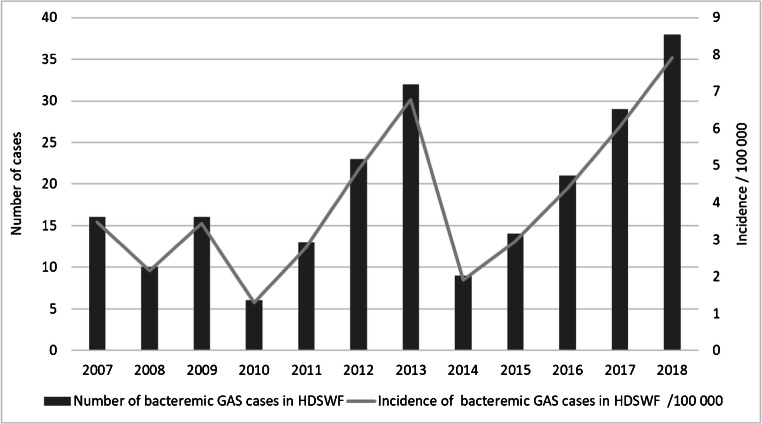
Annual number and incidence/100,000/person-years of bacteremic GAS cases in the HDSWF 2007–2018 as reported in NIDR

In total, 227 GAS bacteremia cases were identified from NIDR. The overall incidence of GAS bacteremia in HDSWF was 3.52/100,000 person-years during the study period, but year-to-year variation was observed with the highest incidence of 7.93/100,000 person-years in 2018. Beside year 2018, high incidence values were also found in 2013 (6.78/100,000 person-years) and 2017 (6.05/100,000 person-years) whereas the incidence was lowest in 2010 (1.29/100,000 person-years) (Fig. 1). Incidences increased over 4-year follow-up periods between 2007 and 2010 (2.59/100,000 person-years), 2011–2014 (4.08/100,000 person-years) and 2015–2018 (5.34/100,000 person-years). The increase was significant in 2011–2014 (IRR = 1.58, 95% CI 1.10–2.27, *p* = 0.013) and 2015–2018 (IRR = 2.07, 95% CI 1.47–2.91, *p* < 0.001) compared with 2007–2010.

After analyzing the SAI data, a total of 212 adult GAS bacteremia cases met the inclusion criteria and were included in the study. The excluded cases were either under 18 years of age or had not been treated in any of the hospitals of the HDSWF. Clinical characteristics of the cases are summarized in Table 1. The most common underlying condition was any atherosclerotic disease 20.8%, but 22.2% of the cases had no underlying disease. Skin and soft tissue infections were the most common clinical manifestations (58.0%). 21.7% of the cases were admitted to intensive care unit (ICU) and 15.6% met the STSS definition. Mortality among all cases in 7 days was 6.6% and in 90 days 13.2%. Case fatality rate (CFR) in STSS was in 7 days 24.2% and in 90 days 27.3%.

**Table 1:** Demographics and clinical characteristics of 212 patients with GAS bacteremia and comparison of patients with and without infectious diseases specialist consultation

Variables	IDSC+ (*n* = 117)	IDSC− (*n* = 71)	*p* value ^a^	NADC (*n* = 24)	Total (212)
Sex: male	56 (47.9)	38 (53.5)	0.452	10 (41.7)	104 (49.1)
Age mean (SD)	56.3 (19.2)	60.9 (19.3)	0.115	72.2 (17.0)	59.6 (19.6)
Healthcare-acquired	19 (16.2)	9 (12.7)	0.506	3 (12.5)	31 (14.6)
Charlson class			0.245		
0	36 (30.8)	14 (19.7)		1 (4.2)	51 (24.1)
1	25 (21.4)	15 (21.1)		6 (25.0)	46 (21.7)
2	20 (17.1)	11 (15.5)		3 (12.5)	34 (16.0)
3	36 (30.8)	31 (43.7)		14 (58.3)	81 (38.2)
Underlying conditions					
Diabetes mellitus	13 (11.1)	16 (22.5)	0.036	6 (25.0)	35 (16.5)
^b^Any atherosclerotic disease	17 (14.5)	17 (23.9)	0.104	10 (41.7)	44 (20.8)
^c^Chronic lung disease	9 (7.7)	4 (5.6)	0.590	3 (12.5)	16 (7.5)
^d^Alcohol abuse	15 (12.8)	9 (12.7))	0.977	2 (8.3)	26 (12.3)
IVDU	9 (7.7)	3 (4.2)	0.346	1 (4.2)	13 (6.1)
^e^Immunosuppr. medication	14 (12.0)	8 (11.3)	0.885	4 (16.7)	26 (12.3)
^f^Any malignancy	11 (9.4)	15 (21.1)	0.024	2 (8.3)	28 (13.2)
^g^No underlying disease	32 (27.4)	14 (19.7)	0.238	1 (4.2)	47 (22.2)
Allergy to penicillin	3 (2.6)	4 (5.6)	0.281	1 (4.2)	8 (3.8)
Clinical manifestation					
^h^SSTI	67 (57.3)	41 (57.7)	0.948	15 (62.5)	123 (58.0)
^i^Necrotizing fasciitis	5 (4.3)	1 (1.4)	0.279	0 (0)	6 (2.8)
Pneumonia	23 (19.7)	18 (25.4)	0.359	5 (20.8)	46 (21.7)
Arthritis	7 (6.0)	3 (4.2)	0.603	3 (12.5)	13 (6.1)
Puerperal sepsis	7 (6.0)	1 (1.4)	0.132	0 (0)	8 (3.8)
Any abscess	17 (14.5)	6 (8.5)	0.217	2 (8.3)	25 (11.8)
Endocarditis	1 (0.9)	0 (0)	0.435	0 (0)	1 (0.5)
Meningitis	2 (1.7)	0 (0)	0.271	0 (0)	2 (0.9)
^j^Other microbiological finding	20 (17.1)	14 (19.7)	0.650	4 (16.7)	38 (17.9)
Disease severity					
^k^Hypotension	43 (36.8)	20 (28.2)	0.033	5 (20.8)	68 (32.1)
^l^ICU admission	34 (29.1)	8(11.3)	0.005	4 (16.7)	46 (21.7)
^m^STSS	22 (18.8)	5 (7.0)	0.026	6 (25.0)	33 (15.6)
^n^DIC	14 (12.0)	2 (2.8)	0.029	3 (12.5)	19 (9.0)
^o^Any surgical intervention	50 (42.7)	19 (26.8)	0.028	1 (4.2)	70 (33.0)
Mortality					
In 7 days	1 (0.9)	3 (4.2)	0.121	10 (41.7)	14 (6.6)
In 30 days	6 (5.1)	6 (8.5)	0.366	11 (45.8)	23 (10.8)
In 90 days	7 (6.0)	8 (11.3)	0.195	13 (54.2)	28 (13.2)

*IDSC* Infectious disease specialist consultation, *NADC* No available data on consultation, *IVDU* Intravenous drug user, *SSTI* Skin and soft tissue infections, *ICU* Intensive care unit, *STSS* Streptococcal toxic shock syndrome, *DIC* Disseminated intravascular coagulation

Data represent: No, (%) of the group

^a^*p* value: comparison of the groups IDSC+ and IDSC− with chi-square test expect with two-sample for age

^b^Includes atherosclerotic cardiovascular or cerebrovascular disease, peripheral arterial disease, and transient ischemic attack diagnosed by neurologist

^c^Includes asthma, chronic obstructive pulmonary disease, lung fibrosis, and chronic hypoventilation

^d^Includes alcohol abuse or alcohol-related medical or social problem according the patient records

^e^Includes use of ≥ 5 mg of prednisolon last 28 d or biological medicine/cytotoxic drugs last 1 year

^f^Includes leukemia, lymphoma, solid tumors, and other cancers

^g^None of chronic diseases above, nor chronic kidney or liver disease, heart failure, inflammatory bowel disease, hypertension, or hypercholesterolemia

^h^Includes erysipelas, cellulitis, infected ulcers, wound infections

^i^Definition: intraoperative diagnosis of NF made by specialists in the fields of surgery

^j^Includes any other culture positive finding than GAS in clinical sample until the fifth day after taking the blood cultures. Excludes probable skin contaminants and mixed flora

^k^Systolic blood pressure < 100 mmHg when the positive blood cultures for GAS were taken

^l^Admission to the ICU during the 48 h after the positive blood cultures for GAS

^m^As defined in the methods section

^n^Definition: clinical picture (bleeding, thrombus, petechiae) and thrombocyte count < 100 × 109/L or abnormal value of INR (international normalized ratio), APTT (activated partial thromboplastin time), TT (thrombin time), or FIDD (fibrin D-dimer)

^o^Includes interventions ranging from wound revision or abscess drainage to amputation

A record of IDSC was found (+) in 117 (55.2%) cases, not found (−) in 71 (33.5%) cases, while in 24 (11.3%) cases, no data on consultation were available. The comparison of the groups IDSC+ and IDSC− is shown in Table 1. The patients with GAS bacteremia who received IDSC were more likely to be admitted to ICU, to have hypotension, STSS and disseminated intravascular coagulation (DIC) and to undergo any surgical intervention. The patients who did not receive IDSC were more likely to have diabetes mellitus and malignancies. Otherwise these two study groups did not differ statistically significantly. There was a slight trend toward lower mortality in IDSC+ group compared with IDSC− group even though it did not reach statistical significance.

### *emm*-type distribution

The *emm*-type distribution of the bacteremic GAS isolates during the study period is summarized in Table 2. The most prevalent *emm* types in the descending order were *emm*28 (a total of 58 cases, 27.4% of all), *emm*1 (42, 19.8%), *emm*89 (40, 18.9%), *emm*12 (18, 8.5%), and *emm*4 (14, 6.6%). During the incidence peak years of 2013 and 2018, the most common *emm* types were *emm*89 (12, 40.0%) and *emm*1 (17, 42.5%). The increase in *emm*1 isolate numbers was observed already in 2016 and 2017 when also the incidence of GAS bacteremia began to increase.

**Table 2:** E*mm*-type distribution of bacteremic GAS isolates (*n* = 212) included in the study

Year	Number of isolates (% of total) per year							
	emm1	emm4	emm12	emm28	emm89	Others	Missing	Total
2007	4 (30.8)	0 (0)	1 (7.70)	2 (15.4)	0 (0)	5 (38.5)	1 (7.7)	13
2008	4 (33.3)	0 (0)	1 (8.3)	4 (33.3)	0 (0)	3 (13.2)	0 (0)	12
2009	1 (7.1)	3 (21.4)	1 (7.1)	2 (14.3)	2 (14.3)	4 (28.6)	1 (7.1)	14
2010	0 (0)	0 (0)	0 (0)	2 (50.0)	0 (0)	2 (50.0)	0 (0)	4
2011	0 (0)	3 (27.3)	1 (9.1)	1 (9.1)	4 (36.4)	2 (18.2)	0 (0)	11
2012	2 (10.0)	1 (5.0)	1 (5.0)	3 (15.0)	10 (50.0)	3 (15.0)	0 (0)	20
2013	3 (10.1)	1 (3.3)	1 (3.3)	8 (26.7)	12 (40.0)	5 (16.7)	0 (0)	30
2014	1 (12.5)	1 (12.5)	0 (0)	0 (0)	3 (37.5)	3 (37.5)	0 (0)	8
2015	0 (0)	0 (0)	0 (0)	6 (42.9)	6 (42.9)	2 (14.3)	0 (0)	14
2016	4 (22.2)	2 (11.1)	5 (27.8)	4 (22.2)	0 (0)	3 (16.7)	0 (0)	18
2017	6 (21.4)	1 (3.6)	6 (21.4)	11 (39.3)	2 (7.1)	2 (7.1)	0 (0)	28
2018	17 (42.5)	2 (5.0)	1 (2.5)	15 (37.5)	1 (2.5)	4 (10.0)	0 (0)	40
total	42 (19.8)	14 (6.6)	18 (8.5)	58 (27.4)	40 (18.9)	38 (17.9)	2 (0.9)	212

The *emm* types observed in ICU treated cases (*n* = 46) in descending order were as follows: *emm*28 (17, 37.0%), *emm*1 (9, 19.6%), *emm*89 (7, 15.2%), *emm*12 (6, 13.0%), and *emm*4 (1, 2.20%) and other *emm* types (6, 13.0%). In cases meeting the STSS criteria (*n* = 33), the most prevalent *emm* types were *emm*28 (9, 27.3%), *emm*1 (8, 24.2%), *emm*89 (5, 15.2%), and *emm*12 (4, 12.1%). The *emm*-type distribution of cases with NF (*n* = 6) was *emm*1 [2], *emm*89 [2], *emm*12 [1], and *emm*6.4 [1].

### Antimicrobial susceptibility

All GAS strains were susceptible to benzylpenicillin. Resistance rate to clindamycin was 4.2% (9 cases) and 2.4% (5 cases) were intermediately sensitive to clindamycin. Erythromycin sensitivity was not investigated in laboratory in years 2011–2016. Erythromycin resistance rate was 1.4% (3 cases in year 2018) and 5.2% (11 cases during the years 2009, 2017, and 2018) were intermediately sensitive to erythromycin. Antimicrobial susceptibility did not differ significantly between study groups.

### Infectious diseases specialist consultation and antibiotic treatment selection

**Fig. 2:**
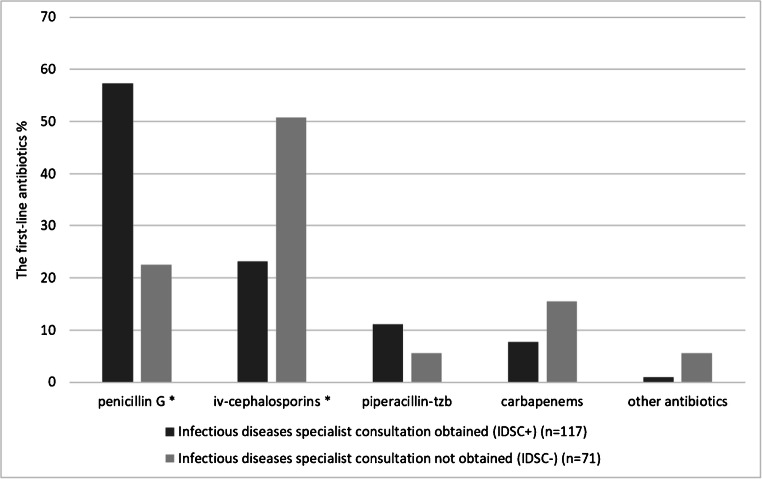
The percentages of the selected first-line antibiotics in the treatment of GAS bacteremia stratified by IDSC. *p < 0.001 (binary logistic regression)

**Fig. 3:**
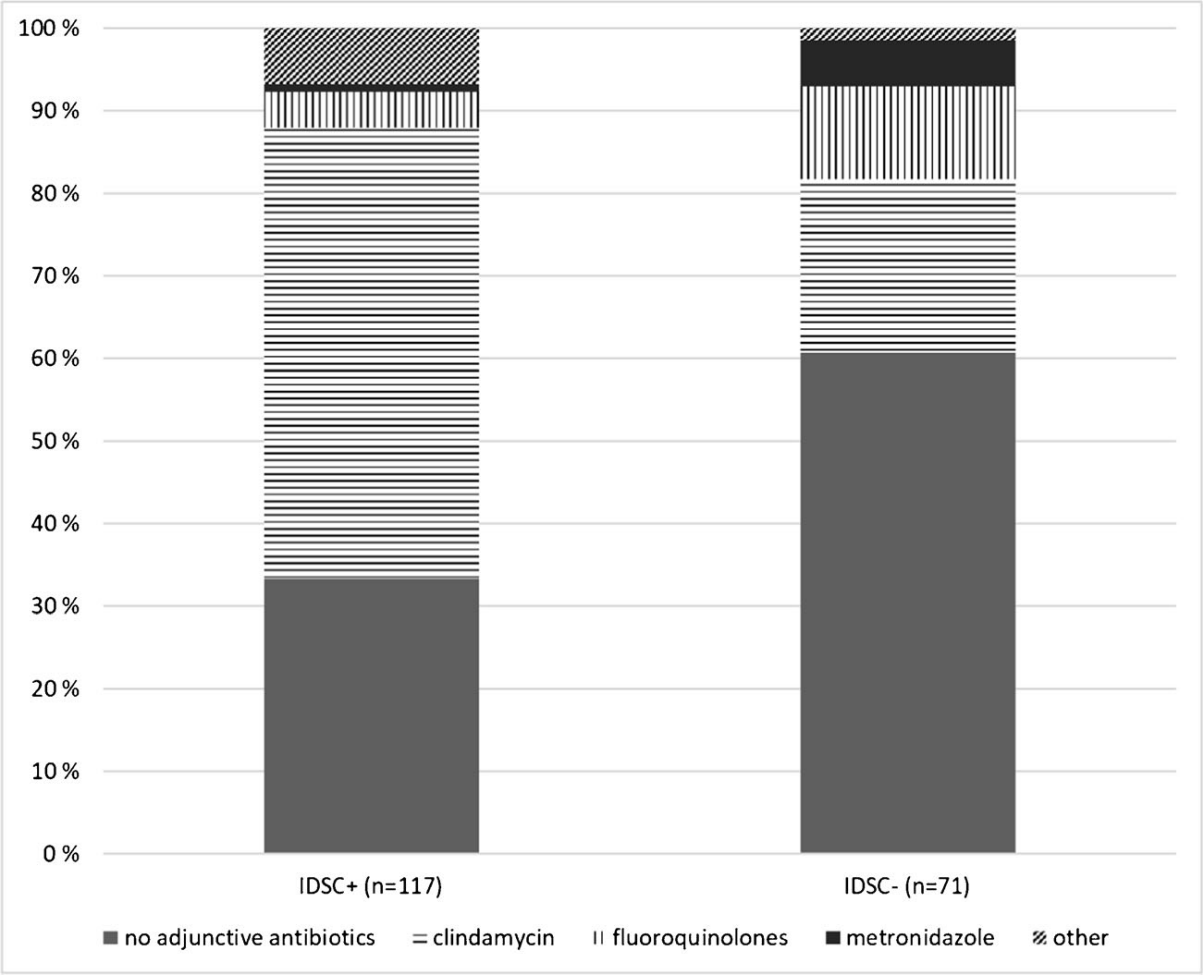
The percentages of the selected adjunctive antibiotics in the two study groups (IDSC+ and IDSC−)

The percentages of the selected first-line antibiotics and adjunctive antibiotics in the treatment of GAS bacteremia stratified by IDSC are shown in Figs. 2 and 3. The most commonly used first-line antibiotics were penicillin G and iv-cephalosporins. The selection of first-line antibiotic differed significantly between the groups IDSC+ and IDSC− (chi-square test, *p* < 0.001). Among IDSC+ cases, 57.3% were on penicillin G treatment whereas in the group IDSC− only 22.5%, respectively (OR = 4.61, 95% CI 2.37–8.97; *p* < 0.001). Among IDSC+ cases only 23.1% were on iv-cephalosporins treatment whereas in the IDSC− group up to 50.7% (OR = 0.29, 95% CI 0.16–0.55; *p* < 0.001). The most commonly used adjunctive antibiotics were clindamycin, fluoroquinolones, and metronidazole. The use of any adjunctive antibiotics was significantly less common in the group IDSC− (39.4%) than IDSC+ (66.7%) (chi-square test, *p* < 0.001). The use of clindamycin as adjunctive antibiotic was more common among IDSC+ cases (54.7%) than IDSC− (21.1%) (OR = 4.51, 95% CI 2.29–8.87; *p* < 0.001).

The antibiotic treatment selection was also viewed in two time periods, 2007–2012 and 2013–2018. In the whole study population, the use of penicillin G as first-line antibiotic increased during the study period from 23.0% (2007–2012) to 47.8% (2013–2018) (*p* < 0.001).

### The antibiotic treatment selections in ICU

Table 3 summarizes the antibiotic treatment selection in the ICU-treated cases compared with the non-ICU-treated cases with or without IDSC. The ICU-treated cases were analyzed as one cohort because 34 cases (81%) obtained IDSC. Cases with NADC (see Introduction; definitions) were excluded from this analysis. In 12 cases, the antibiotic treatment included 3 antibiotics. Eight of these 12 were ICU-treated. In 8 cases, the third antibiotic was regarded as clindamycin while the second antibiotic was regarded as either a carbapenem or a fluoroquinolone combined to the first-line antibiotic penicillin during the data collection. In ICU-treated cases, the use of clindamycin and carbapenems were more prevalent than in non-ICU-treated.

**Table 3:** Comparison of the antibiotic treatment selections in ICU-treated and non-ICU-treated cases with or without IDSC

Antibiotic selection	ICU treated (*n* = 42)	Non-ICU-treated IDSC+ (*n* = 83)	OR (95% CI)	*p* value ^a^	Non-ICU-treated IDSC− (*n* = 63)	OR (95% CI)	*p* value ^b^
Penicillin G as FA	21 (50.0)	49 (59.0)	1.44 (0.68–3.04)	0.337	13 (20.6)	0.26 (0.11–0.61)	0.002
Iv-cephalosporins as FA	5 (11.9)	23 (27.7)	2.84 (0.99–8.11)	0.052	35 (55.6)	9.25 (3.21–26.64)	< 0.001
Piperacillin-tzb as FA	6 (14.3)	7 (8.4)	0.55 (0.17–1.76)	0.316	4 (6.3)	0.41 (0.11–1.54)	0.185
Carbapenems as FA	10 (23.8)	3 (3.6)	0.12 (0.031–0.47)	0.002	7 (11.1)	0.40 (0.14–1.15)	0.090
Clindamycin use in all combinations ^c^	36 (85.7)	43 (51.8)	0.18 (0.07–0.47)	< 0.001	8 (12.7)	0.02 (0.01–0.08)	<0.001

*IDSC* infectious disease specialist consultation, *ICU* intensive care unit, *FA* first-line antibiotics

Data represents: No, (%) of the group

^a^*p* value: comparison of the groups ICU-treated and non-ICU-treated IDSC+ with logistic regression

^b^*p* value: comparison of the groups ICU-treated and non-ICU treated IDSC− with logistic regression

^c^Clindamycin in all combinations includes all reported cases with clindamycin use (even as third antibiotic)

## Discussion

This study shows an overview of GAS bacteremia in one hospital district in Finland during 2007–2018. We observed the alarmingly high incidence of GAS bacteremia in 2018, the increasing trend in incidence during the study period, and that IDSC increased the use of narrow-spectrum beta-lactam antibiotics, but, on the other hand, increased also the use of clindamycin as adjunctive antibiotic.

Our observed overall incidence of GAS bacteremia is comparable with earlier studies from Finland with iGAS incidence ranging from 2.46/100,000 to 3.6/100,000, but the peak-incidence is higher than ever before reported from Finland [4, 5, 18]. The criteria for reporting iGAS to the NIDR have remained unaltered throughout the years. The overall incidence is also comparable with iGAS studies conducted in other western countries [18–21]. The incidence of iGAS infections increased in 2018 also in whole Finland to the highest level of 6.74/100,000 ever since 1995 when NIDR was established [14]. However, as there is no common international definition on an iGAS case, and the surveillance systems differ between countries, exact comparisons on iGAS rates between countries and studies need to be done with caution. With a broader case definition (a case with blood/CSF GAS isolate, or with a positive GAS culture from any sterile site, or with clinical signs of iGAS and a GAS positive culture from a nonsterile site), we would have recorded even more cases.

The observed *emm-*type distribution in this study is, in general, similar to earlier studies in Finland and Europe [4, 5, 22]. The proportion of *emm*1 was high in the years 2007–2008 and increased again since 2016. In 2018, *emm*1 was the most prevalent *emm* type in our HD as well as also in the whole country, thus most probably causing the epidemic wave [23]. Similarly, Lynskey et al. have reported a dominant new *emm*1 *Streptococcus pyogenes* lineage emerging in England from year 2015 onwards [8]. Among severe GAS bacteremia cases with ICU admission, STSS and NF *emm*1 was a common *emm* type. This is in line with earlier studies where *emm*1 has been associated with death and severe manifestations such as STSS and NF [2, 21, 22]. During the study period 2007–2018, *emm*28 has been among the three most common *emm* types in Finland [23]. In the current study, *emm*28 was the most prevalent *emm* type. In our study, a surprisingly high proportion of ICU-treated and STSS cases were caused by *emm*28. In a previous large study from Europe, only 9% of STSS cases were caused by *emm*28 [22]. Latronico et al. reported a new *emm*89 clone emerging in 2009 and spreading rapidly in Finland [24]. This was also observed in HDSWF in years 2011–2013.

The prevalence of diabetes and malignancies as underlying condition was comparable with other studies from Northern Europe [25–27]. Even higher prevalence of diabetes (29.3%) and association with iGAS disease and death were observed in a recent study from the USA [28]. The rates of STSS cases in our study (15.6%) were comparable to rates 10.0 to 19.0% that were observed in other studies from western countries [18, 25, 26, 28, 29]. However, in our study, the CFR for STSS in 90 days (27.3%) was lower than in other studies with CFR 38–44% [18, 21]. The study population with STSS in this study was, of course, limited.

In the current study, IDSC increased the use of penicillin G as first-line antibiotic whereas the lack of IDSC increased the use of broad-spectrum beta-lactam antibiotics. We also discovered that the use of penicillin G in whole study population increased significantly during the later study period (2012–2018). This observation is important because the global concern of antimicrobial resistance calls for action. The first generally available antimicrobial guide of the HDSWF was published in the end of 2012. The amount of infectious diseases specialists has increased during the later study period (2012–2018) in HDSWF. These may explain, at least partly, the improved adherence to the rational use of antibiotics. Seppälä et al have earlier shown that nationwide guidance on antibiotic use resulted in reduction in the use of macrolide antibiotics for outpatient respiratory and skin infections and a significant decline in the frequency of erythromycin resistance among group A streptococci [30]. However, the latter may be confounded by variation of the prevalent *emm* types, as shown by Smit et al. [5].

To our knowledge there are no earlier studies regarding the role of IDSC in the antibiotic treatment selection of GAS bacteremia. However, many previous studies have demonstrated the benefits of IDSC in *Staphylococcus aureus* infections [31–33].

According to literature, the use of clindamycin as adjunctive therapy for non-STSS iGAS infections is elusive whereas the benefit is observed in cases with STSS and NF [11–13]. Brindle et al. showed in a clinical trial that the addition of clindamycin to flucloxacillin did not improve outcome in limb cellulitis but doubled the likelihood of diarrhea [34]. Some international guidelines recommend considering addition of clindamycin to beta-lactam therapy in GAS bacteremia without shock or other organ failure [35]. The rationale behind this suggestion is based on a small retrospective study in children as well as on experimental mouse model data [36, 37]. There seems to be conflicting opinions on the indications of the combined clindamycin use internationally. In the current study, the use of clindamycin as adjunctive antibiotic was more common than the prevalence of STSS or NF, and it occurred mainly in the group IDSC+. Among ICU-treated cases, the clindamycin use was most prevalent. However, the use of clindamycin as adjunctive therapy for non-severe iGAS infections lacks evidence of clinical trials with adult patients and is known to cause high risk for adverse consequences and the use should thus be avoided in these cases.

Any other culture positive finding than GAS in a clinical sample was equally present in the study groups. Thus, polymicrobial findings do not explain the excessive use of cephalosporins in the group IDSC− nor the excessive clindamycin use in the group IDSC+.

The strength of the current study is that the clinical data were gathered by reading all the electronic patient records by an infectious diseases specialist. The electronic patient records offered detailed data on, e.g., antimicrobial therapy. This study has also limitations. The study population is small and represents patients only from one hospital district in Finland. Regarding antimicrobial therapy and its side-effects, we did not study the length of clindamycin use or the prevalence of diarrhea. On average, the consultations regarding the antibiotic selections in bacteremic patients, even done by phone, are well documented in HDSWF, but as the study method is retrospective, there is a possibility that undocumented IDSC took also place. Infectious diseases specialist visits regularly in ICU and takes care of the patient with the physicians of the ICU. These routine consultations taking place during the rounds are not necessarily always documented. Thus, the proportion of IDSC in ICU may be even higher than reported in this study.

In conclusion, GAS caused bacteremias with alarmingly high incidence in one hospital district in Finland in 2018. Narrow-spectrum beta-lactam antibiotics were chosen, but excess clindamycin as adjunctive antibiotic was used, if IDSC took place. This highlights the importance of availability of IDSC when aiming for more rational antibiotic use but calls for improved practice among infectious diseases specialists by avoiding combination therapy with clindamycin in non-severe iGAS infections.

## Data Availability

The datasets generated during the current study are not publicly available as they contain health related data but limited datasets (without any identifiable, person-related data) are available from the corresponding author on reasonable request.
